# Life-Threatening Aspiration Pneumonia as a Rare Complication of Postoperative Nausea and Vomiting (PONV) in a Young Patient Following Cardiac Surgery: A Case Report

**DOI:** 10.7759/cureus.83254

**Published:** 2025-04-30

**Authors:** Hideyuki Kuratomi, Masafumi Idei, Shunsuke Takaki, Nobuyuki Yokoyama, Masashi Yokose

**Affiliations:** 1 Department of Critical Care Medicine, Yokohama City University Hospital, Yokohama, JPN

**Keywords:** airway management, anesthetic management, aspiration pneumonia, cardiac surgery, postoperative nausea and vomiting

## Abstract

We report a young cardiac surgery patient who developed severe aspiration pneumonia due to postoperative nausea and vomiting (PONV) despite prophylactic and therapeutic administration of multiple antiemetic agents.

A 19-year-old woman underwent corrective surgery for partial anomalous pulmonary venous connection. On postoperative day (POD) 1, she was extubated after receiving dexamethasone (6.6 mg), droperidol (1 mg), and granisetron (1 mg) for PONV prophylaxis. Despite metoclopramide, nausea persisted. On POD 2, severe aspiration pneumonia followed massive vomiting, worsening oxygenation. Thus, emergency reintubation was required. The patient remained on mechanical ventilation until POD 8 and in the intensive care unit until POD 12.

We reported a rare case of severe aspiration pneumonia due to PONV. Young cardiac surgery patients, including those with congenital heart disease, are at a high PONV risk, making perioperative prevention and treatment crucial.

## Introduction

Postoperative nausea and vomiting (PONV) is a common postoperative complication, occurring in approximately 30% of surgical patients and up to 80% of high-risk patients [1,2]. PONV significantly impacts patient comfort and satisfaction and can delay postoperative recovery. Moreover, it has been associated with complications such as dehydration, electrolyte imbalance, increased risk of wound dehiscence, unanticipated hospital admissions, and overall increased healthcare costs. However, cases in which PONV leads to life-threatening complications such as severe aspiration or critical hypoxemia are exceedingly rare.

The pathophysiology of PONV is complex and involves the activation of the vomiting center, a neural network located in the medulla oblongata that includes the reticular formation and the nucleus tractus solitarius. Afferent input to the vomiting center is received through several pathways. One originates from the chemoreceptor trigger zone (CTZ) located in the area postrema, which detects emetogenic substances in the blood and cerebrospinal fluid. Another pathway involves visceral afferent fibers traveling via the vagus nerve from the gastrointestinal mucosa. Mechanoreceptors located within the gastrointestinal mucosa, such as stretch and pressure receptors, detect distension of the intestinal wall caused by increased luminal contents. These sensory signals are transmitted primarily via the vagus nerve to the vomiting center, where they may trigger nausea and the emetic reflex, and are often stimulated by surgical manipulation. Additional input from the vestibular system, transmitted through the vestibulocochlear nerve (cranial nerve VIII), plays a role particularly in motion-induced nausea. These multiple pathways converge on the vomiting center, where integration of emetogenic signals ultimately triggers the nausea and vomiting response.

Risk factors for PONV are categorized as patient-, anesthesia-, and surgery-related factors [3]. Patient-related risk factors include female sex, nonsmoking status, and younger age [4]. Anesthesia-related risk factors include the use of volatile anesthetics and intraoperative or postoperative opioids, both of which are known to stimulate emetogenic pathways through multiple mechanisms. Volatile anesthetics can directly activate the CTZ in the area postrema by enhancing dopaminergic and serotonergic neurotransmission, while also sensitizing the vestibular system. This vestibular sensitization may reduce lower esophageal sphincter tone, thereby increasing the risk of gastric reflux. Opioids, on the other hand, not only stimulate the CTZ but also delay gastric emptying and increase gastrointestinal stasis, thereby enhancing vagal afferent signaling to the vomiting center. These combined effects significantly elevate the risk of PONV. Surgery-related risk factors include laparoscopic, gynecological, and strabismus surgeries [5,6]. These procedures often involve visceral irritation or manipulation and increased intra-abdominal pressure, which activate vagal afferents and enhance emetogenic signaling to the vomiting center.

Although rare, severe complications resulting from PONV can have significant clinical consequences. In this report, we present a case of a young patient who developed severe aspiration pneumonia following extubation after cardiac surgery, despite receiving multiple prophylactic and therapeutic antiemetic agents. The patient's postoperative course was complicated by respiratory failure requiring prolonged ventilatory support and an extended stay in the intensive care unit (ICU). This case underscores the potential severity of PONV-related complications and highlights the need for comprehensive, individualized perioperative management strategies, particularly in high-risk surgical populations such as those undergoing cardiac procedures.

## Case presentation

The patient was a 19-year-old non-smoking woman (height: 163 cm and weight: 50 kg) with a history of motion sickness. The patient was diagnosed with an atrial septal defect at a one-month-old health checkup, which closed spontaneously. When she turned 16, an abnormality was detected on a school chest radiograph. Contrast-enhanced computed tomography (CT) revealed partial anomalous pulmonary venous return with the right lower pulmonary vein draining into the azygos vein, leading to the decision of surgical repair. Thus, the patient underwent intracardiac repair under cardiopulmonary bypass and was admitted to the ICU while intubated. The surgery lasted 11 hours and 44 minutes, with a total anesthesia time of 13 hours and 48 minutes. Remifentanil was administered intraoperatively at a rate of 0.25 to 0.5 μg/kg/min, in addition to a total fentanyl dose of 2000 μg. After the surgery, sedation was maintained with continuous infusions of fentanyl (0.2-0.4 μg/kg/hr), propofol (1-3 mg/kg/hr), and dexmedetomidine (0.3-1 μg/kg/hr). On postoperative day (POD) 1, the patient met the criteria for a spontaneous awakening trial and the spontaneous breathing trial, leading to the decision to extubate. Because the patient was considered at high risk for PONV, dexamethasone (6.6 mg), granisetron (1 mg), and droperidol (1 mg) were intravenously administered before extubation. After extubation, the patient developed nausea and received additional doses of metoclopramide (10 mg) and droperidol (1.25 mg); however, the patient’s symptoms persisted. The patient’s oxygen saturation (SpO2) remained above 95% on 2-3 L/min of nasal cannula oxygen, and there were no signs of hoarseness, dysphagia, abdominal pain, or abnormal intestinal gas patterns on abdominal radiography. As the patient experienced mild thirst and requested some water, the patient was allowed to drink a few sips; however, due to ongoing nausea, we withheld the resumption of oral food intake.

**Figure 1:**
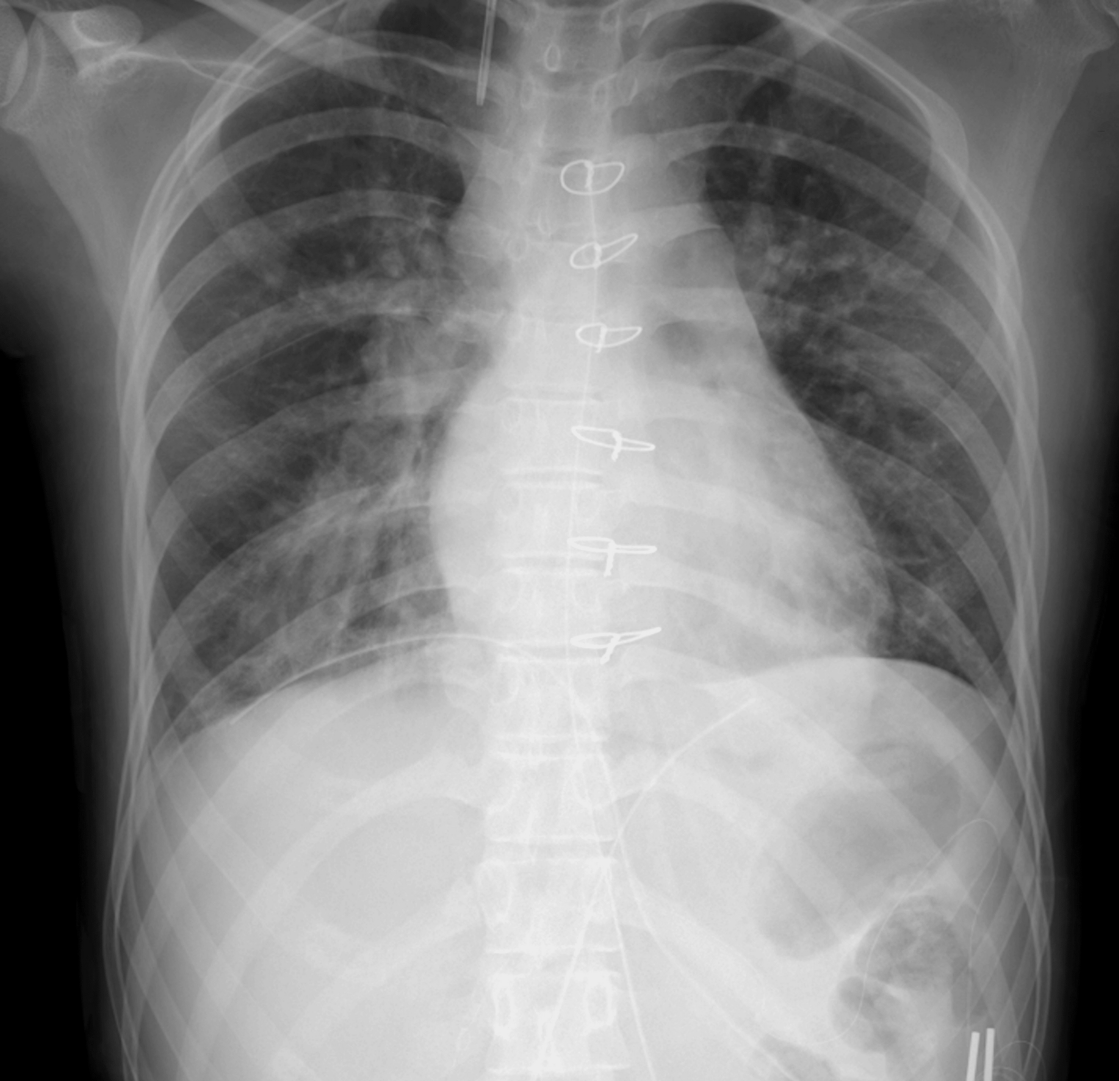
Chest radiograph obtained before extubation

**Figure 2:**
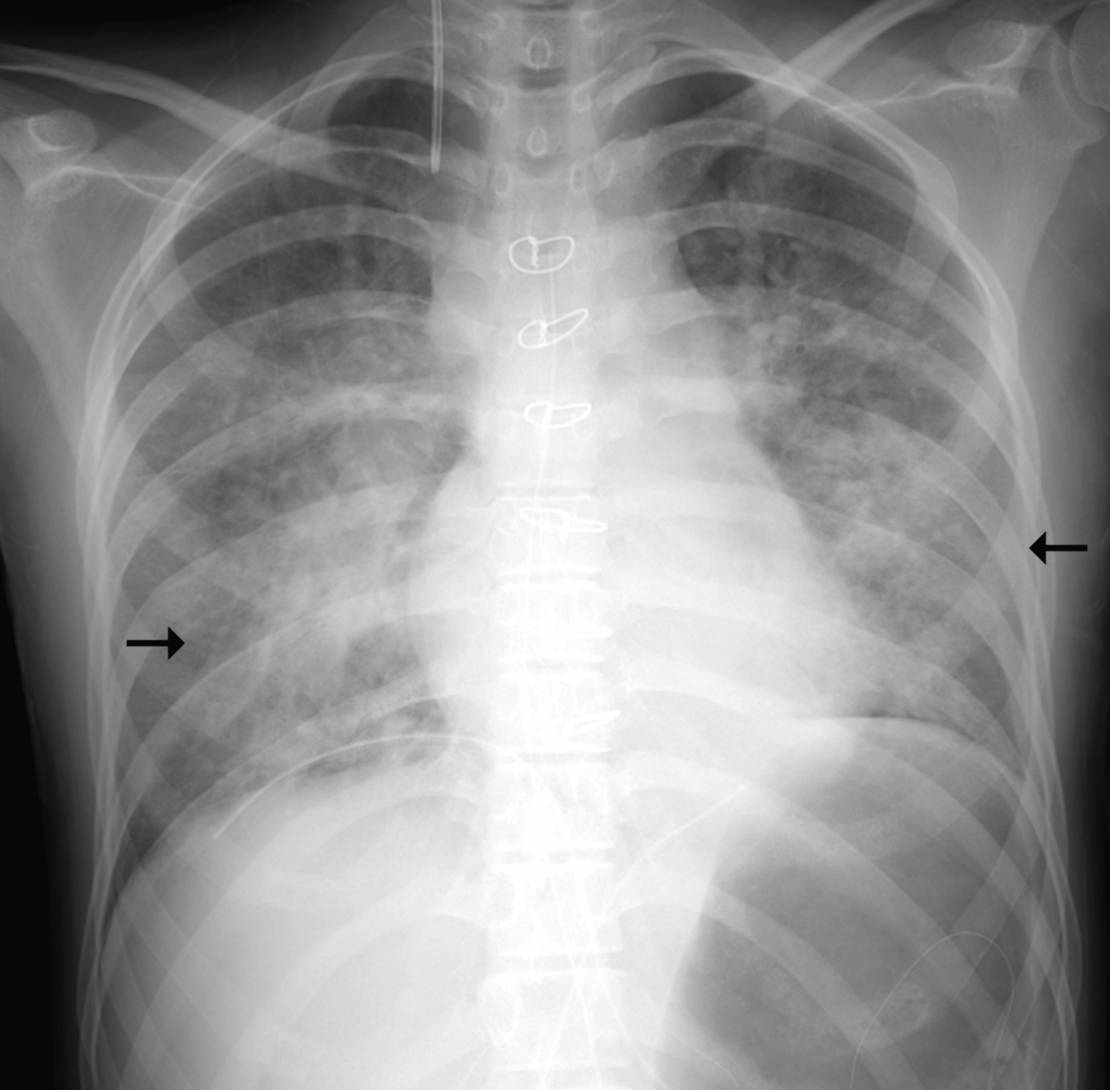
Chest radiograph obtained after aspiration showed diffuse bilateral consolidation (arrows)

**Figure 3:**
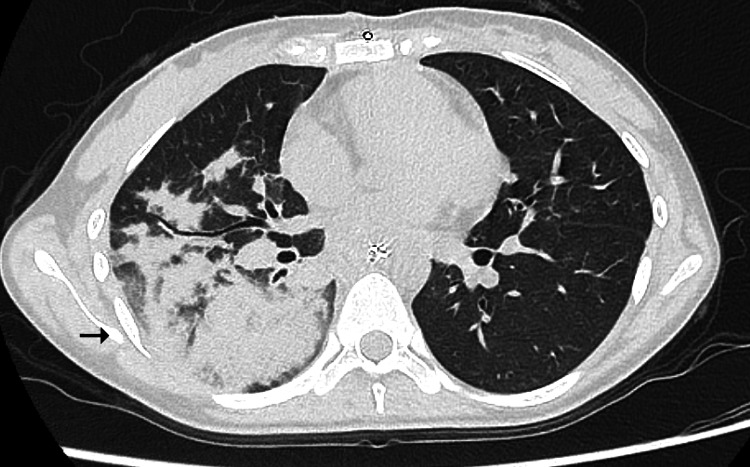
Computed tomography (CT) scan obtained after reintubation revealed massive consolidation predominantly in the right lower lobe (arrow)

On POD 2, although intermittent intravenous administration of 10 mg metoclopramide provided transient relief, the patient's nausea remained unresolved. The patient experienced massive vomiting at night, leading to a rapid decline in SpO2 from 96% to 70-79% on 3 L/min of nasal cannula oxygen. Arterial blood gas analysis revealed the partial pressure of oxygen to be 39 mmHg and the partial pressure of carbon dioxide to be 47 mmHg, indicating critical hypoxemia. The patient was promptly placed in a sitting position, and thorough suctioning of the oral and pharyngeal secretions was performed. Immediate positive pressure ventilation with 100% oxygen was initiated using a Jackson-Rees circuit; however, the SpO2 did not improve adequately and ranged in the 80s. Chest radiography and CT revealed extensive consolidation (Figure 1, Figure 2, Figure 3), and the patient was diagnosed with severe aspiration pneumonia, necessitating emergency reintubation and mechanical ventilation.

Post-reintubation transesophageal echocardiography showed no evidence of cardiac dysfunction or pulmonary vein stenosis. CT of the brain and abdomen and blood tests ruled out other potential causes of nausea and vomiting such as intracranial pathology, mechanical bowel obstruction, and electrolyte imbalance. The patient was treated with ampicillin/sulbactam (3 g intravenously every six hours) for pneumonia. To promote gastrointestinal motility, she also received prokinetic agents, including the traditional Japanese herbal medicines daikenchuto (15 g/day orally) and rikkunshito (15 g/day orally), both of which are known for their prokinetic properties. Additionally, she was administered pantothenol, a pantothenic acid derivative (250 mg intravenously every 12 hours), and naldemedine tosylate (0.2 mg/day orally). Pain management was maintained with remifentanil administered at a dosage of 0.05-0.1 μg/kg/min, and mechanical ventilation was continued.

On POD 8, the patient’s oxygenation improved under pressure support ventilation with a positive end-expiratory pressure (PEEP) of 5 cm H₂O, pressure support (PS) of 5 cm H₂O, and a fraction of inspired oxygen (FiO₂) of 0.3, resulting in a partial pressure of arterial oxygen (PaO₂) of 139 mmHg and a respiratory rate of 18 breaths per minute. The spontaneous awakening trial confirmed the resolution of nausea. Metoclopramide (10 mg), granisetron (1 mg), and droperidol (1.25 mg) were administered intravenously before extubation. After extubation, nausea was managed using granisetron (1 mg) and droperidol (1.25 mg), as needed. The patient's respiratory condition remained stable and her PONV symptoms gradually resolved. She was discharged from the ICU on POD 12. The patient was discharged from our institution on postoperative day (POD) 49, ambulating independently and without any neurological or physical sequelae, following completion of a therapeutic anticoagulation regimen for a pulmonary vein thrombus.

## Discussion

This case illustrates a rare but severe complication of PONV - life-threatening aspiration pneumonia requiring prolonged mechanical ventilation - in a young cardiac surgery patient, despite guideline-based antiemetic prophylaxis. 

PONV is a common postoperative complication, occurring in approximately 30% of surgical patients and up to 80% of high-risk patients [1,2]. 

The 2020 American guidelines recommend 5-HT3 receptor antagonists (granisetron and ondansetron), dopamine receptor antagonists (droperidol and metoclopramide), and corticosteroids (dexamethasone) as primary prophylactic and therapeutic agents for PONV. Additional options that are not available in Japan include neurokinin receptor antagonists (aprepitant) and dopamine receptor antagonists (amisulpride) [1,7,8]. These agents act via different mechanisms and are often used alone or in combination. The timing of administration varies, depending on the pharmacokinetics of each drug. When PONV develops despite prophylaxis, the administration of an antiemetic with a different mechanism of action within six hours of the previous dose is recommended [9].

Although PONV is a relatively common complication, cases in which it leads to life-threatening complications are rare. There have been reports of PONV-associated Boerhaave syndrome, requiring emergency surgery [10], and aspiration pneumonia, necessitating rehospitalization after discharge. However, cases in which PONV-induced vomiting leads to critical hypoxemia and a prolonged ICU stay, such as ours, are scarce.

Cardiac surgery is linked to multiple risk factors for PONV (Table 1), including a long operation duration, opioid use, and impaired gastrointestinal motility due to surgical stress and cardiopulmonary bypass. Previous reports have indicated severe PONV in pediatric patients with congenital heart disease undergoing cardiac surgery or catheter-based interventions. Postoperative PONV complications include aspiration pneumonia, wound dehiscence, postoperative hemorrhage, and hemodynamic instability. After cardiac surgery, 3-9% of patients develop dysphagia, with recurrent laryngeal nerve paralysis occurring in 0.7-2% of adults and 0.1-0.5% of pediatric patients, increasing aspiration risk [11,12]. Additionally, vomiting-induced increases in intra-abdominal pressure and sympathetic stimulation may strain tissue sutures and vascular anastomoses, thereby increasing the risk of wound dehiscence and postoperative hemorrhage. Vagal stimulation associated with PONV can also cause bradycardia and hypotension, further destabilizing circulation.

**Table 1:** Risk factors and complications of postoperative nausea and vomiting (PONV) in cardiac surgery. Created by the authors with reference to references [1,5].

Types of anesthesia	Risk factors
Risk factors for PONV in general anesthesia	Female sex
	Nonsmoking
	History of PONV or motion sickness
	Younger age
	Use of inhaled anesthetics
Risk factors for PONV characteristic of cardiac surgery	Prolonged surgery
	Intraoperative and postoperative opioid use
	Decreased intestinal peristalsis
Complications from PONV after cardiac surgery	Blood pressure variation
	Bradycardia due to vagal reflex
	Tachycardia due to stress
	Rebleeding
	Wound dehiscence
	Pneumonia
	Hindrance to getting out of bed

In retrospect, several aspects of perioperative management may have contributed to the development of this complication and warrant critical reflection. First, the intraoperative administration of 2000 μg of fentanyl, although justified by the need to supplement remifentanil in response to surgical invasiveness and potential opioid tolerance, may have been excessive and contributed to postoperative nausea. A more judicious opioid-sparing strategy, including greater reliance on remifentanil - an ultra-short-acting opioid with a favorable pharmacokinetic profile - might have been preferable, particularly in the context of high PONV risk. In addition, a multimodal analgesia approach incorporating regional anesthesia techniques, such as paravertebral blocks, intercostal nerve blocks, or erector spinae plane blocks, could have further mitigated opioid requirements, potentially enhancing patient outcomes. Furthermore, continuous fentanyl infusion was maintained until the initial extubation, which may have prolonged opioid exposure unnecessarily. In contrast, remifentanil was appropriately used for analgesia following reintubation and discontinued at the time of extubation to avoid residual opioid effects.

Second, although the volume was minimal, the resumption of oral water intake prior to complete resolution of nausea may have been premature. In high-risk patients, even small amounts of oral intake should be withheld until symptoms fully subside to reduce the risk of vomiting and aspiration. Greater awareness of the potential severity of PONV following cardiac surgery might have prompted a more cautious and preventative approach in this case.

Our patient had multiple risk factors for PONV, including female sex, young age, nonsmoking status, history of motion sickness, prolonged surgery, and opioid use. Despite the prophylactic administration of multiple antiemetics, the patient developed PONV, which led to severe aspiration pneumonia. In addition to these risk factors, gastrointestinal hypomotility due to surgical stress and cardiopulmonary bypass may contribute to vomiting via the vagal and chemoreceptor trigger zone-mediated pathways. Following reintubation, the addition of prokinetic agents, including daikenchuto, rikkunshito, pantothenol, and naldemedine tosylate, may have provided additional benefit in resolving the patient’s PONV symptoms.

## Conclusions

We report a rare case of severe aspiration pneumonia due to PONV despite guideline-based prophylaxis. Young cardiac surgery patients have a high risk of PONV and a greater likelihood of developing severe complications, such as aspiration pneumonia. The perioperative care team should maintain a shared awareness of each patient’s individual risk for PONV and recognize the potential severity of PONV-related complications. Furthermore, perioperative prevention and management of PONV should involve strict adherence to established clinical guidelines. However, it is important to recognize that guideline-adherent care may be insufficient in patients with multiple risk factors, thereby necessitating heightened vigilance and a more individualized management approach to optimize outcomes. Particular attention should be given to minimizing the use of opioids with residual effects, such as fentanyl, by limiting both the dosage and duration of administration to the minimum necessary.
